# Supplementary material for: Determination of Reactive Oxygen or Nitrogen Species and Novel Volatile Organic Compounds in the Defense Responses of Tomato Plants against Botrytis cinerea Induced by Trichoderma virens TRS 106
Source: Cells. 2022 Sep 29;11(19):3051. doi: 10.3390/cells11193051 (PMC9563596; doi:10.3390/cells11193051)
Supplement: Supplementary file 1 [file cells-11-03051-s001.zip › cells-1883625-supplementary.pdf]

Figure S1.

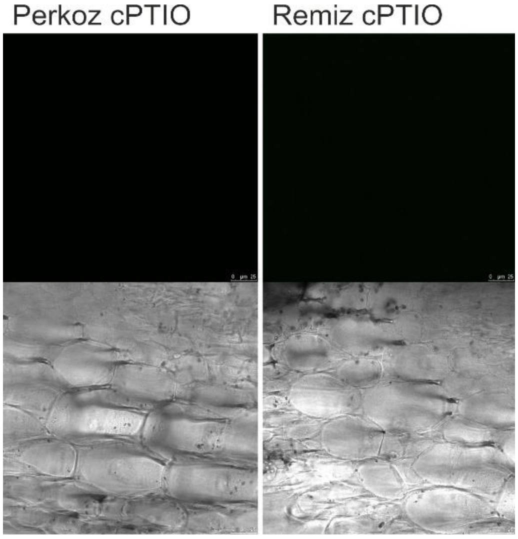

Table S1. Effect of TRS 106 on tomato growth parameters. Values represent the means + SE from four independent experiments with four replicates each (n=16). Separately for each parameter, differences between varieties within a given treatment were analyzed with Student's t-test and marked using letters A and B. Separately for each variety, differences between TRS 106 and control plants were analyzed with Student's t-test and marked using asterisk symbol with the following manner (* P < 0.05; ** P < 0.01; *** P < 0.001). Influence of the variety (Perkoz and Remiz) and TRS treatment and their interaction on the investigated traits was checked with two-way ANOVA. Abbreviations: TRS 106, plants grown in the soil with *T. virens* TRS 106 spores; FW, fresh weight; DW, dry weight.

**Parameter**	**Perkoz**		**Remiz**	
	**Control**	**TRS 106**	**Control**	**TRS 106**
Tomato shoot FW [g]	10.86 ± 1.25 B	15.44 ± 1.13 B***	9.94 ± 0.05 A	13.74 ± 2.48 A***
Tomato shoot DW [g]	1.64±0.13 A	1.91±0.13 A***	2.64±0.53 B	3.57±0.57 B***
Tomato roots FW [g]	2.46±0.41 A	2.88±0.33 A**	3.18±0.60 B	3.41±0.50 B
Tomato roots DW [g]	0.24±0.05 A	0.30±0.04 A**	0.28±0.06 A	0.30±0.05 A
Tomato shoot height [cm]	47.14±4.58 A	50.24±2.56 A**	49.88±6.08 A	51.69±3.30 A*
Tomato shoot thickness [mm]	3.97±0.34 A	4.97±0.29 A***	4.31±0.48 A	4.84±0.68 A*
Total tomato leaves area [cm^2]	215.78±15.82 A	271.18±29.23 A***	337.95±24.68 B	381.83±50.14 B**

Table S2. Results of two-way ANOVA investigating effects of studied treatments on the tested parameters of the studied tomato varieties. Abbreviations: FW, fresh weight; DW, dry weight.

(a)

Dependent variable	*F* value and significance		
	Variety (C; df = 1)	Treatment (T; df = 1)	V x T (df = 1)
**Growth**			
Tomato shoot FW [g]	11.89***	121.32***	1.05
Tomato shoot DW [g]	178.47***	36.99***	11.14***
Tomato roots FW [g]	28.10***	7.56**	0.68
Tomato roots DW [g]	2.26	7.25***	2.68
Tomato shoot height [cm]	3.28	16.83***	0.16
Tomato shoot thickness [mm]	0.87	42.42***	3.98
Total tomato leaves area [cm^2]	205.15***	37.31***	0.50
**Disease area on leaves [%]**	103.93***	83.67***	0.74

(b)

Dependent variable	*F* value and significance		
	Variety (C; df = 1)	Treatment (T; df = 3)	V x T (df = 3)
**ROS metabolism**			
$O_2^{\cdot -}$ content [A g^{-1} FW]	13.29***	5.92*	0.51
H_2O_2 content [nmol g^{-1} FW]	13.97***	21.51***	1.70
SOD activity [U mg^{-1} protein]	0.78	6.92***	27.40***
**RNS metabolism**			
NO content [nmol g^{-1} FW]	121.74***	36.02***	6.47***
$ONOO^-$ content [nmol g^{-1} FW]	912.58***	20.48***	32.36***
SNO content [nmol mg^{-1} protein]	41.33***	21.12***	24.22***
GSNOR activity [U mg^{-1} protein]	10.09**	3.43*	1.94
**VOC**			
**Total GLV and derivatives**			
2-Hexenal	964.48***	6.86***	5.94***
3-Hexenal	2535.60***	91.79***	73.39***
Hexanal	1985.14***	32.25***	35.37***
2,4-Hexadienal	36.04***	1.36 n.s.	12.63***
4-Ethyl-2-hexynal	371.40***	371.40***	371.40***
1-Hexanol	3801.61***	3.54*	4.40**
2-Hexanol	313.44***	90.44***	74.76***
4-Methyl-3-hexanol	7711.78***	906.54***	906.54***
2-Ethyl-1-hexanol	15.17**	2.98 n.s.	1.38n.s.
2-Hexen-1-ol	343.63***	37.68***	15.40***
2-Hexyn-1-ol	3464.79***	3794.83***	3938.98***
1,5-Hexadien-3-ol	304.51***	2229.28***	394.01***
**Aromatic compounds**			
Benzaldehyde	1907.62***	253.05***	252.81***
4-Ethylbenzaldehyde	553.79***	13.97***	10.95***
4-Methylbenzaldehyde	36.65***	126.15***	81.21***
2-Hydroxyacetophenone	3669.71***	1361.65***	1237.12***

Acetophenone	130.50***	35.78***	55.26***
Methyl salicylate	224.70***	84.13***	26.59***
Ethyl salicylate	1175.87***	536.53***	543.22***
Isoamyl salicylate	212.22***	187.69***	187.69***
4-Hepten-2-yl salicylate	76.90***	124.50***	100.46***
Phenol	3669.71***	1361.65***	1237.12***
2-Methylphenol	861.91***	29.83***	18.91***
2-Methoxyphenol	29.33***	52.29***	32.76***
